# *GNAQ/GNA11* Mosaicism Is Associated with Abnormal Serum Calcium Indices and Microvascular Neurocalcification

**DOI:** 10.1016/j.jid.2023.09.008

**Published:** 2024-04

**Authors:** Nicole Knöpfel, Davide Zecchin, Hanna Richardson, Satyamaanasa Polubothu, Sara Barberan-Martin, Thomas Cullup, Karolina Gholam, Simon Heales, Steve Krywawych, Pablo López-Balboa, Noreen Muwanga-Nanyonjo, Olumide Ogunbiyi, Clinda Puvirajasinghe, Lea Solman, Katherine Swarbrick, Samira B. Syed, Zubair Tahir, Martin M. Tisdall, Jeremy Allgrove, Alexander D. Chesover, Sarah E. Aylett, Thomas S. Jacques, Fadil M. Hannan, Ulrike Löbel, Robert K. Semple, Rajesh V. Thakker, Veronica A. Kinsler

**Affiliations:** 1 Mosaicism and Precision Medicine Laboratory, Francis Crick Institute, London, United Kingdom; 2 Genetics and Genomic Medicine, UCL GOS Institute of Child Health, London, United Kingdom; 3 Department of Paediatric Dermatology, Great Ormond St Hospital for Children, London, United Kingdom; 4 Neurodisability, Great Ormond St Hospital for Children, London, United Kingdom; 5 North Thames Genomic Laboratory Hub, Levels 4-6, Barclay House, Great Ormond St Hospital for Children NHS Foundation Trust, London, United Kingdom; 6 Department of Chemical Pathology NIHR BRC, Great Ormond St Hospital for Children NHS Foundation Trust, London, United Kingdom; 7 Department of Histopathology, Great Ormond St Hospital for Children NHS Foundation Trust, London, United Kingdom; 8 Paediatric Neurosurgery, Great Ormond St Hospital for Children NHS Foundation Trust, London, United Kingdom; 9 Endocrinology, Great Ormond St Hospital for Children NHS Foundation Trust, London, United Kingdom; 10 Developmental Biology and Cancer Programme, UCL GOS Institute of Child Health, London, United Kingdom; 11 Nuffield Department of Women's & Reproductive Health, University of Oxford, Oxford, United Kingdom; 12 Radiology, Great Ormond St Hospital for Children, London, United Kingdom; 13 Centre for Cardiovascular Science, Queen’s Medical Research Institute, University of Edinburgh, Edinburgh, United Kingdom; 14 Academic Endocrine Unit, Radcliffe Department of Medicine, University of Oxford, Oxford, United Kingdom; 15 National Institute for Health Research Oxford Biomedical Research Centre; Oxford, United Kingdom

## Abstract

Mosaic mutations in genes *GNAQ* or *GNA11* lead to a spectrum of diseases including Sturge-Weber syndrome and phakomatosis pigmentovascularis with dermal melanocytosis. The pathognomonic finding of localized “tramlining” on plain skull radiography, representing medium-sized neurovascular calcification and associated with postnatal neurological deterioration, led us to study calcium metabolism in a cohort of 42 children. In this study, we find that 74% of patients had at least one abnormal measurement of calcium metabolism, the commonest being moderately low serum ionized calcium (41%) or high parathyroid hormone (17%). Lower levels of ionized calcium even within the normal range were significantly associated with seizures, and with specific antiepileptics despite normal vitamin D levels. Successive measurements documented substantial intrapersonal fluctuation in indices over time, and DEXA scans were normal in patients with hypocalcemia. Neurohistology from epilepsy surgery in five patients revealed not only intravascular, but perivascular and intraparenchymal mineral deposition and intraparenchymal microvascular disease in addition to previously reported findings. Neuroradiology review clearly demonstrated progressive calcium deposition in individuals over time. These findings and those of the adjoining paper suggest that calcium deposition in the brain of patients with *GNAQ/GNA11* mosaicism may not be a nonspecific sign of damage as was previously thought, but may instead reflect the central postnatal pathological process in this disease spectrum.

## Introduction

**Figure 1:**
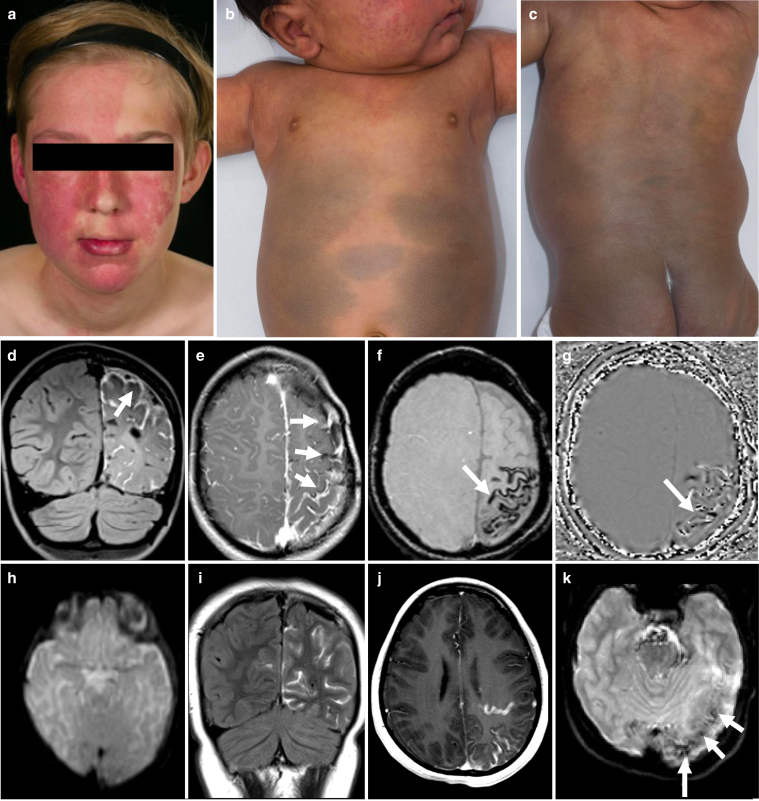
**Phenotypic and radiological features in *GNAQ/GNA11* mosaicism.** (**a**) Clinical features of a patient with SWS with a capillary malformation of the face involving the critical forehead area (Waelchli et al., 2014), associated with glaucoma in the right eye. This patient had hypocalcemia with low levels of ionized and total calcium and increased PTH. (**b** and **c**) Clinical features of a patient with PPV-DM exhibiting a capillary malformation (with naevus anaemicus) on the face, upper trunk, and extensive areas of dermal melanocytosis. (**d** and **e**) Contrast enhanced fluid-attenuated inversion recovery and T1-weighted magnetic resonance images of a patient with SWS show left frontal, parietal, and occipital pial angiomatosis (vascular malformation, arrows). (**f**) Susceptibility-weighted imaging (SWI) and (**g**) SWI phase map depicting vascular calcifications (arrow). This patient had low levels of ionized calcium and total calcium with PTH in the normal range. (**h**) Axial DWI b0 image does not show calcifications on the baseline magnetic resonance imaging study. (**i**–**k**), images from 8-years later. (**i**) Coronal fluid-attenuated inversion recovery after administration of a gadolinium-based contrast agent shows left parietal and occipital (and some contralateral) sulcal enhancement in keeping with pial angioma. (**j**) Axial postcontrast T1-weighted image depicts the sulcal enhancement and a prominent draining vein. (**k**) DWI b0 image at follow-up shows foci of increased susceptibility which are suggestive of calcification. Patients and/or parents/guardians provided written consent for the publication of images. Ca/Cr, calcium to creatinine ratio; PTH, parathyroid hormone.

With the discovery of many of the causal genes, complex phenotypic classifications of mosaic disorders affecting the skin have now been grouped together as disease spectra*.* The spectrum of *GNAQ*/*GNA11* mosaicism essentially encompasses variable combinations of vascular and/or pigmentary abnormalities variably affecting the skin, eyes, and brain (Figure 1). Well-defined phenotypes include Sturge-Weber syndrome (SWS, purely vascular) and phakomatosis pigmentovascularis with dermal melanocytosis (PPV-DM, vascular and pigmentary). For a full description of the phenotypic variability in this spectrum we refer the reader to previous publications (Polubothu et al, 2020; Shirley et al, 2013; Sliepka et al, 2019; Thomas et al, 2016). The neurovascular abnormalities in *GNAQ/GNA11* mosaicism present with seizures, neurodevelopmental impairment, headaches, and stroke-like episodes (Comi, 2007), which progress postnatally. The original, classical, and pathognomonic finding associated with neurovascular disease is neurovascular calcification, first seen on plain-skull radiography in which the parallel lines of calcification of affected blood vessel walls were described as “tramlining” (Figure 1f and g). This tramlining was in fact visualization of calcified medium-sized veins within the leptomeninges. Despite its pathognomonic nature, and in particular its absence from other mosaic vascular malformations affecting the brain, this feature has been considered to be a nonspecific marker of tissue damage (Boström et al, 1993). With the advent of increasingly advanced imaging techniques, neuropathology in *GNAQ/GNA11* mosaicism has concluded that slow blood flow through the leptomeningeal vascular malformations is responsible for underlying parenchymal hypoxia and hence neurological deterioration over time (Kelley et al, 2005; Lin et al, 2006; Pilli et al, 2017). Interestingly however, both the degree of intracranial calcification and the degree of venous hypoperfusion on radiological studies have been correlated with neurological symptoms (Kelley et al, 2005; Lin et al, 2006; Pilli et al, 2017). These facts suggested to us that the calcification may be central to the process of neurological deterioration, and may be a clue to the localized vascular biological abnormalities.

Calcium is a critical component of transmembrane and intracellular signaling (Bootman et al, 2001). As such, serum calcium levels are tightly controlled by homeostatic mechanisms involving parathyroid hormone (PTH) and vitamin D (Goltzman et al, 2018), and so critical are the levels that maintenance of normal serum calcium levels will take priority over, for example, bone health. Disease states such as inherited monogenic diseases affecting calcium metabolism, such as familial hypocalciuric hypercalcemia and pseudohypoparathyroidism types 1A and 1B, are well-documented to lead to intravascular calcification (Iwase et al, 2019; Pollak et al, 1993). Mosaic disorders however, although monogenic, only affect some parts of the body, and in this regard could conceivably behave more like multiple tumor foci than a genetic disease. We, therefore, hypothesized that the known neurovascular calcification could be related to abnormal calcium fluxes in and around foci of affected blood vessels, and that this could potentially affect serum calcium levels to some degree. We further hypothesized that microvascular calcification could be an undocumented aspect of disease that could lead to brain tissue hypoxia, independent of leptomeningeal involvement. As a result, we undertook a large cross-sectional study of children with diagnoses in the *GNAQ/GNA11* mosaicism spectrum, to determine their calcium metabolic profile in addition to deep clinical phenotyping and offering genotyping. In parallel, we reviewed all available neuroimaging and neurohistology, particularly to characterize the neurocalcification.

## Results

### SWS/PPV-DM cohort has classical neuroradiological and clinical progression and genotypic profile

Forty-two patients were recruited, 21 females, 31 with SWS, 2 with extensive cutaneous capillary malformations and 9 with PPV-DM (Figure 1a–c). Mean and median ages were 7.62 (SEM 0.76) and 8 years respectively (range 0.2–16.1). Phenotypic, genotypic, and results data are summarized in Table 1. A key finding was a mean and median age of onset of seizures of 0.98 years (SEM 0.25) and 0.63 (range 0–5.92) respectively, confirming the postnatal neurological deterioration described in previous studies. Intracranial calcifications were detectable on imaging in 50% of patients (Figure 1f and g). Neurovascular calcification was clearly demonstrated to develop over time where multiple scans were available from the same patient (Figure 1h–k). Genotyping was accepted by 29 patients and results were representative of previous cohort publications (Jordan et al, 2020; Polubothu et al, 2020; Shirley et al, 2013). The pathogenic variants underlying the clinical diagnoses were identified as follows: SWS caused by a mosaic variant in *GNAQ* c.548G>A, p.(R183Q) in 17 patients; widespread CM caused by *GNA11* c.547C>T, p.(R183C) in one; PPV-DM caused by *GNAQ* c.548G>A, p.(R183Q) in three and by *GNA11* c.547C>T, p.(R183C) in another five. Three patients (two SWS and one PPV-DM) were double-wildtype (WT).

**Table 1:** Patient Deep Phenotyping, Genotyping and Serum Calcium Metabolic Profile

Patient No.	Sex	Age, years	Genotype	Cutaneous features	Neurological features	Ophthalmological features	Other clinical findings	Age at most recent brain MRI, years	Most recent brain MRI findings	Ionized calcium	PTH
1	F	3.8	*GNAQ* c.548G>A, p.R183Q	CM face (right, including forehead)	Seizures, stroke-like episodes, and headaches. Normal development	Left increased IOP and choroidal hemangioma	-	3.6	LA left frontal and temporal lobes associated with cortical calcifications. Underdevelopment of left hemispheric superficial cortical veins, enlargement of left deep medullary veins and osteohypertrophy on the left. Generalized parenchymal volume loss in the left cerebral hemisphere. Prominent left cerebellar vessels	Normal	Normal
2	M	3.1	*GNAQ* and *GNA11* WT	No vascular or pigmentary lesions	Seizures, stroke-like episodes, left hemiplegia. Normal development	Normal	-	2.9	LA right parietal lobe associated with subjacent parenchymal volume loss and gyriform calcifications. Slight enlargement of the right choroid plexus	Normal	Normal
3	M	16.1	*GNAQ* c.548G>A, p.R183Q	CM face (bilateral, including forehead) and neck	Intellectual disability, autism, and ADHD	Left glaucoma and bilateral choroidal hemangiomas	Joint hypermobility and muscle weakness	14.8	LA right parietal, occipital and temporal lobes. Prominent and atypical veins in the right hemisphere and right cerebellum. Progressive volume loss of cerebellar hemispheres	Normal	Low
4	F	4.0	-	CM right forehead	None	Right glaucoma	-	1.2	LA right occipital and temporal lobes. Abnormal venous drainage in the DMVs territory. Abnormal vessels on the right sylvian fissure and choroid plexus suggestive of DVA	Normal	Normal
5	M	15.9	-	CM face (bilateral, including forehead), neck, upper trunk, and lower limbs	Seizures, left hemiplegia, severe intellectual disability, autism, and language disorder	Bilateral glaucoma	Left hip subluxation and valgus deformity of left knee (with overlying vascular lesions on the skin)	13.1	LA right hemisphere associated with cerebral atrophy and gyriform calcifications. Left cerebellar leptomeningeal angiomatosis	Low	Normal
6	M	2.7	-	CM face (bilateral, including forehead)	None	Left buphthalmos, left glaucoma and cloudy cornea	-	0.03	Prominent left cortical and leptomeningeal vessels	Normal	Normal
7	F	11.0	*GNAQ* c.548G>A, p.R183Q	CM face (bilateral, including forehead) and scalp	Seizures, left hemiplegia, headaches, intellectual disability, autism, ADHD and language disorder	Bilateral glaucoma	Scoliosis and leg length discrepancy (with no overlying vascular lesions)	1.9	LA right hemisphere with progression of subjacent cerebral atrophy. LA left frontal lobe, insular cortex and mid brain	-	Normal
8	M	1.9	*GNAQ* c.548G>A, p.R183Q	CM face (bilateral, including forehead) right upper limb and right abdomen associated with overgrowth	Seizures, stroke-like episodes and left hemiplegia. Normal development	Normal	Coronal hypospadias and right hydrocele	1.7	LA right frontal and parietal lobes associated with calcifications on the frontal lobe. Prominent draining DMVs frontal lobe and left insula	Normal	Normal
9	F	11.8	*GNAQ* c.548G>A, p.R183Q	CM face (bilateral, including forehead), neck and upper trunk	Seizures, right hemiplegia, headaches, intellectual disability, autism, ADHD and language disorder	Left glaucoma	Scoliosis. Cervicothoracic lipoma	9.6	LA left hemisphere with subjacent parenchymal volume loss and multiple draining veins. Enhancement of the brainstem.	Low	Normal
10	M	11.6	*GNAQ* c.548G>A, p.R183Q	CM left forehead	Seizures, right hemiplegia, headaches, intellectual disability, autism, ADHD and language disorder	Normal	Recurrent epistaxis	7.7	LA left hemisphere with subjacent parenchymal volume loss. Increased draining veins within the ventricles, cortical signal change and enhancement	Normal	Normal
11	M	15.0	-	Bony prominence left forehead. No vascular or pigmentary lesions on skin	Seizures, headaches, anxiety. Normal development	Right homonymous hemianopia	-	15.0	LA left temporal, parietal and occipital lobes with subjacent parenchymal volume loss and gyriform calcifications. Progression of thickening and bone expansion of the diploic spaces of the left frontal bone	Low	Normal
12	M	2.4	-	CM face (left, including forehead)	Seizures, developmental impairment and social communication difficulties	Left glaucoma and left choroidal hemangioma	-	0.3	LA left parietal and occipital lobes with subjacent parenchymal volume loss. Choroid plexus asymmetry	-	Normal
13	F	9.2	*GNAQ* c.548G>A, p.R183Q	CM face (bilateral, including forehead), trunk and lower limb	Seizures, cerebral palsy of 4 limbs and intellectual disability	Bilateral glaucoma	Microcephaly	6.5	LA both frontal and parietal lobes and left temporal lobe associated with calcifications. Thickening of the skull and prominent deep cerebral DVAs (predominantly on the right)	Normal	Normal
14	F	9.4	*GNAQ* c.548G>A, p.R183Q	CM left forehead	Seizures, right hemiplegia, intellectual disability, autism, ADHD and language disorder	Left increased IOP	-	9.4	LA left frontal, parietal and occipital lobes with subjacent parenchymal volume loss and gyriform calcifications	Low	Normal
15	M	11.8	*GNAQ* c.548G>A, p.R183Q	CM face (bilateral, including forehead), scalp, trunk and limbs associated with overgrowth	Seizures, cerebral palsy of 4 limbs and intellectual disability	Bilateral glaucoma	Scoliosis. Left hip dysplasia and dislocation (with overlying vascular lesions on the skin)	9.6	No imaging available	Normal	Normal
16	M	1.4	-	CM forehead (bilateral) and scalp	Seizures, right hemiplegia and developmental impairment	Bilateral increased IOP		1.6	LA both parietal lobes and right frontal lobe with calcifications. Prominent deep veins along lateral ventricles, left hippocampus, midbrain and the midline. Bilateral enlargement of choroid plexus	-	-
17	F	4.0	-	CM face (left, including forehead), scalp and neck	None	Visual field defect	-	0.4	LA left occipital lobe. Enlargement of left choroid plexus	Normal	Normal
18	F	0.7	*GNAQ* c.548G>A, p.R183Q	CM right forehead	Seizures, right hemiplegia and developmental impairment	Normal	-	0.7	LA right parietal and temporal lobes associated with subjacent parenchymal volume loss and gyriform calcifications. Enlargement of right choroid plexus	Normal	High
19	M	10.9	*GNAQ* c.548G>A, p.R183Q	CM face (left, including forehead), trunk and limbs associated with overgrowth	Seizures, stroke-like episodes, intellectual disability, autism, ADHD and language disorder	Left glaucoma	Myasthenia gravis	10.8	LA parietal, temporal and occipital lobes. Encephalomalacia related to left hemispheric temporo-parietal-occipital disconnection and anterior temporal lobectomy	Low	Normal
20	M	10.8	-	CM face (bilateral, including forehead), neck and upper trunk. Café-au-lait macule neck	Seizures and headaches. Normal development	Left glaucoma	Obesity, acanthosis nigricans, gynecomastia and isolated adrenarche. Hypomineralis-ed dentition	7.8	LA left temporal, parietal and occipital lobes with subjacent parenchymal volume loss and gyriform calcifications. Large transmantle vein on the left extending to enlarged choroid plexus and DVA	Low	Normal
21	M	5.5	*GNAQ* and *GNA11* WT	CM face (left, including forehead), neck and upper trunk	Stroke-like episodes. Social communication difficulties	Normal	-	3.7	LA left cerebellar hemisphere associated with a large DVA	Low	Normal
22	M	11.2	*GNAQ* c.548G>A, p.R183Q	CM right forehead	Seizures, left hemiplegia, intellectual disability, autism and ADHD	Right glaucoma and left homonymous hemianopia	Scoliosis	6.8	LA right hemisphere with calcifications. Signs of right functional hemispherectomy with shrinkage of the right cerebral hemisphere and mature cystic leukomalacia.	Low	Normal
23	M	13.7	*GNAQ* c.548G>A, p.R183Q	CM face (bilateral, including forehead) and neck	Seizures, stroke-like episodes, headaches, intellectual disability and language disorder	Right glaucoma	-	10.5	LA right frontal lobe with gyriform calcifications	Low	High
24	F	10.3	*GNAQ* c.548G>A, p.R183Q	CM face (bilateral, including forehead), neck, upper trunk and limb associated with overgrowth	Headaches, intellectual disability and language disorder	Left glaucoma	-	7.1	LA right temporal lobe, occipital lobes, splenium of corpus callosum, midbrain and pons. Multiple DVAs in supra- and infra-tentorial compartments	Normal	Normal
25	F	9.6	-	CM face (bilateral, including forehead), neck, buttock and lower limb associated with overgrowth	Seizures, left hemiplegia, intellectual disability, autism, language disorder and dyslexia	Normal	-	6.4	LA right parietal and occipital lobes with calcifications. Signs of disconnection surgery and likely residual connection medially in the right parietal lobe	Low	Normal
26	F	6.00	*GNAQ* c.548G>A, p.R183Q	CM face (bilateral, including forehead), neck, trunk and limbs associated with overgrowth	Seizures, left hemiplegia, intellectual disability and autism	Right glaucoma	-	5.9	LA right cerebral hemisphere. Enlargement of right choroid plexus	Normal	Normal
27	F	2.4	*GNAQ* c.548G>A, p.R183Q	CM right forehead	Seizures. Normal development	Normal	-	2.4	LA right parietal, temporal, and occipital lobes with calcifications. Enlargement of right choroid plexus	Low	High
28	F	15.4	-	CM face (left, including forehead) and scalp	Seizures, right hemiplegia, headaches, language disorder, intellectual disability	Left glaucoma and left choroidal hemangioma	-	11.2	LA left frontal, parietal and temporal lobes with cerebral atrophy and gyriform calcifications. Enlargement of left choroid plexus. Anomalous draining veins on the right cerebral hemisphere	Low	Normal
29	F	1.3	-	CM face (midline, including forehead)	None	Normal	-	1.2	LA both frontal, parietal and occipital lobes associated with prominent medullary veins		
30	M	5.9	*GNA11* c.547C>T, p.R183C	CM with naevus anaemicus face (right, including forehead), trunk and limbs associated with undergrowth	Learning difficulties, Autism	Normal	Leg length discrepancy (with overlying vascular lesions on the skin)	0.9	Small ill-defined foci of signal abnormality in the right caudate nucleus and the adjacent internal capsule, with some associated focal ex vacuo dilatation of the right frontal horn	Low	Normal
31	M	4.3	*GNAQ* c.548G>A, p.R183Q	CM face (right, including forehead)	Seizures, left hemiplegia, stroke-like episodes, intellectual disability and language disorder	Right glaucoma, right choroidal hemangioma	Microcephaly	2.8	Bilateral supra- and infratentorial leptomeningeal angiomatosis with right frontal and temporal lobes relatively spared. Enlargement of right choroid plexus. Bilateral gyriform calcifications in the right temporoparietal and left parietal lobes. Anomalous draining veins more prominent	Low	High
32^1^	F	10.5	*GNA11* c.547C>T, p.R183C	CM face (bilateral, no forehead involvement), trunk and limbs associated with undergrowth	None	Right iris heterochromia. Right increased IOP	Leg length discrepancy (with overlying vascular lesions on the skin)	6.7	Normal brain findings	Normal	Normal
33^1^	F	8.4	*GNA11* c.547C>T, p.R183C	CM and extensive dermal melanocytosis face (including forehead), trunk and limbs associated with overgrowth	Seizures, right hemiplegia, intellectual disability and language disorder	Bilateral glaucoma		8.7	LA left hemisphere, right frontal lobe and cerebellar hemisphere. Cortical calcifications left cerebral cortex and right frontal lobe	Normal	Normal
34^1^	F	7.6	*GNAQ* c.548G>A, p.R183Q	CM trunk and limbs associated with overgrowth. Dermal melanocytosis lower back and café-au-lait macule lower limb	None	Conjunctival melanosis	-	3.2	Normal brain findings	Normal	Normal
35^1^	F	14.3	*GNAQ* and *GNA11* WT	CM trunk and limbs. Dermal melanocytosis lower back. Café-au-lait macule, lower limb	None	Normal	Recurrent epistaxis	-	Brain MRI not performed^2^	-	High
36	F	6.3	*GNA11* c.547C>T, p.R183C	CM with naevus anaemicus face (bilateral, including forehead), trunk and limbs associated with overgrowth. Café-au-lait macular pigmentation, trunk	Language difficulties	Normal	-	6.0	Small cortical hyperintensity in right parietal lobe, without diffusion restriction or abnormal enhancement and overlying calvarial thinning. No other intracranial abnormalities	Low	Normal
37^1^	M	3.8	*GNA11* c.547C>T, p.R183C	CM with naevus anaemicus face (right, including forehead), upper trunk and limbs. Extensive dermal melanocytosis, trunk and limbs	None	Bilateral glaucoma	-	0.7	Suspected calcifications, lateral wall of left lateral ventricle. No focal abnormality or areas of abnormal contrast enhancement	-	Normal
38^1^	M	12.6	*GNAQ* c.548G>A, p.R183Q	CM face (no forehead involvement), upper trunk and limbs associated with overgrowth. Naevus anaemicus left foot. Extensive dermal melanocytosis, trunk and limbs	ADHD	Normal	-	-	Brain MRI not performed^2^	Low	Normal
39	F	2.0	*GNAQ* c.548G>A, p.R183Q	CM upper trunk and upper limb. Extensive dermal melanocytosis, trunk and limbs	None	Normal	-	-	Brain MRI not performed^2^	Normal	Normal
40	M	9.8	*GNA11* c.547C>T, p.R183C	CM trunk and limbs associated with naevus anaemicus and undergrowth. Café-au-lait macular pigmentation neck	Intellectual disability, autism and language disorder	Normal	Leg length discrepancy (with overlying vascular lesions on the skin)	3.1	Normal brain findings	Normal	High
41	F	1.5	-	CM trunk and lower limbs associated with naevus anaemicus and overgrowth.	None	Normal	-	-	Brain MRI not performed^2^	Normal	High
42	M	0.2	-	CM face (bilateral, including forehead), trunk and upper limb	None	Right glaucoma	-	0.02	LA right frontoparietal and occipital lobes. Enlargement of right choroid plexus	Normal	Normal

Abbreviations: ADHD, attention deficit hyperactivity disorder; CM, capillary malformation; DMV, deep medullary vein; DVA, developmental venous anomaly; F, female; IOP, intraocular pressure; LA, leptomeningeal angiomatosis; M, male; MRI, magnetic resonance imaging; PTH, parathyroid hormone; WT, wild-type.

Ionized calcium levels were corrected to pH. Pediatric range references of ionized calcium: 1.15–1.41 mmol/L (<2 years), 1.19–1.37 mmol/L (2–5 years), 1.22–1.31 mmol/L (5–15 years). PTH reference range: 0.7–5.6 pmol/L. Results refer to the first measurements.

According to published guidelines (Waelchli et al, 2014), we currently do not perform MRI/MRA in the absence of vascular lesions on the forehead area unless there are neurological symptoms/signs.

1 indicates a patient previously reported in Polubothu et al. (2020).

2 According to published guidelines (Waelchli et al, 2014), we currently do not perform MRI/MRA in the absence of vascular lesions on the forehead area and neurological symptoms.

### Patients with SWS/PPV have fluctuating levels of serum ionized calcium with normal 25-hydroxy-vitamin D levels

**Figure 2:**
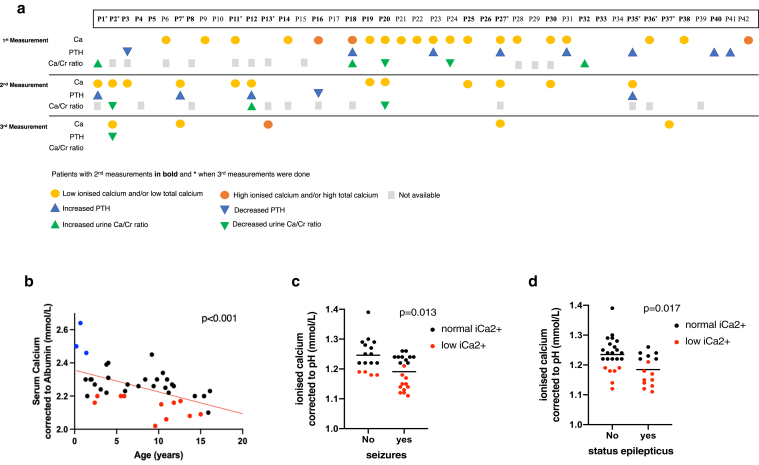
**Fluctuating abnormalities of calcium metabolic profile in patients with *GNAQ/GNA11* mosaicism and association with seizures and status epilepticus.** (**a**) Graphical representation of abnormal results in calcium profiling investigations in the cohort of patients at different time points, demonstrating intra and interpatient variability typical in mosaic disease. Where no coloured marker is shown the result was normal. (**b**) Significant correlation between age and serum calcium corrected to albumin from whole patient cohort. Red and blue dots correspond to serum calcium measurements below or above normal range, respectively, black dots are normal measurements. Linear regression analysis showed a statistically significant negative correlation (*P* < .001). (**c**) Correlation between occurrence of seizures and serum ionized calcium corrected to pH from the patients’ cohort. The scatter plot shows the mean of the two groups, and red dots correspond to ionized calcium measurements below normal range. Linear regression analysis showed a statistically significant correlation (*P* = .013), independent of the effect of age. (**d**) Correlation between status epilepticus and serum ionized calcium corrected to pH in the patients’ cohort. The scatter plot shows the mean of the two groups, and red dots correspond to ionized calcium measurements below normal range. Linear regression analysis showed statistically significant correlation (*P* = .017), independent of the effect of age.

**Supplementary Figure S1:**
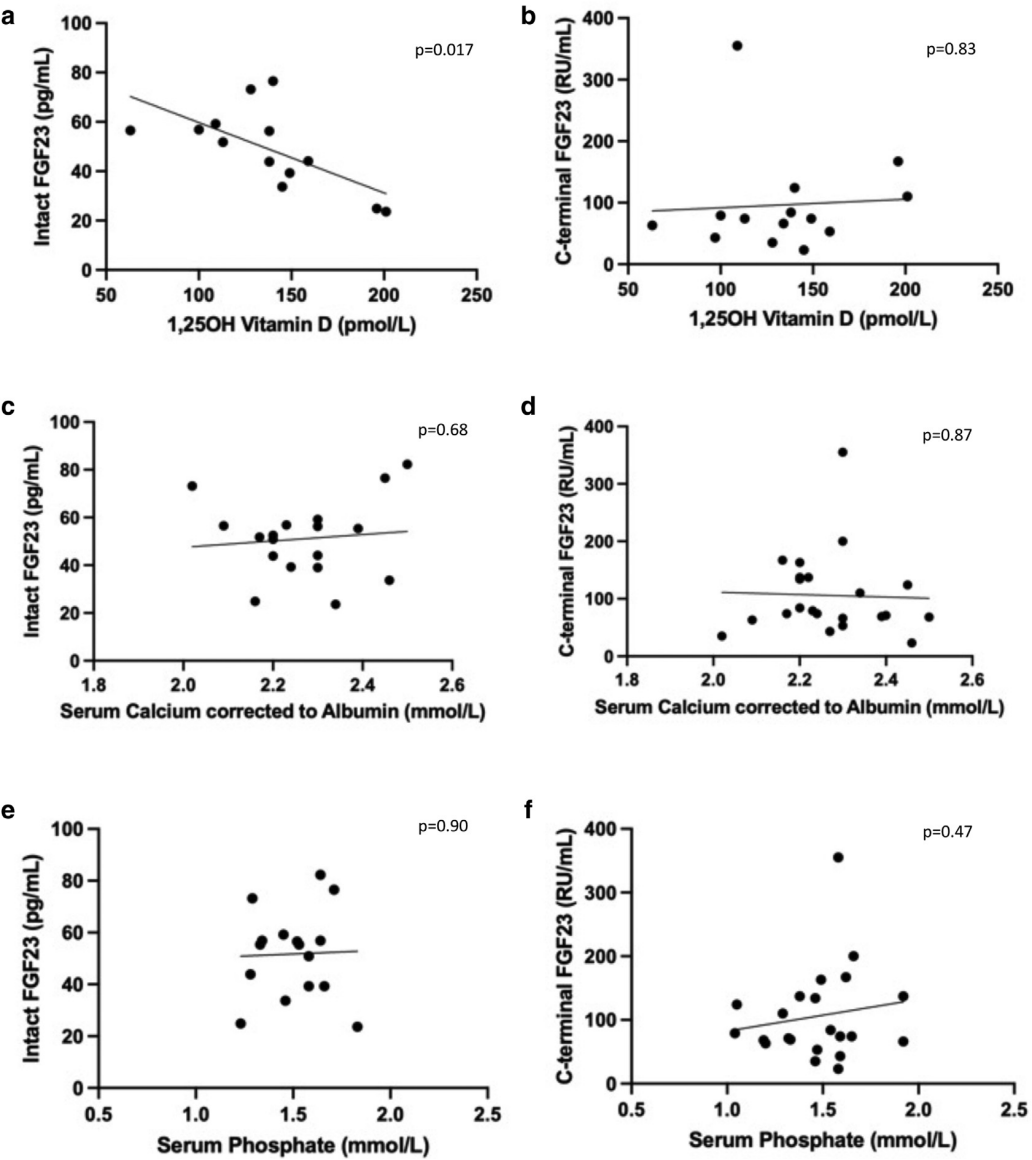
**FGF23 (intact molecule and C-terminal) correlation to 1,25-hydroxy vitamin D, serum calcium corrected to albumin and serum phosphate.** Note the opposite relationships between 1,25-hydroxyvitamin D and C-terminal FGF23 and its biologically active form intact FGF23. (**a** and **b**) Correlation between intact and C-terminal FGF23 and 1,25-hydroxyvitamin D. (**c** and **d**) Correlation between intact and C-terminal FGF23 and albumin-corrected serum calcium. (**e** and **f**) Correlation between intact and C-terminal FGF23 and serum phosphate. Statistical correlations were performed in Prism as detailed in the methods. FGF23, fibroblast growth factor 23; 1,25OH, 1,25-hydroxy.

All calcium metabolism–related parameters were measured in the same diagnostic laboratory using age- and sex-adjusted reference intervals from the UK normal population. Three patients had low 25-hydroxy-vitamin D on first measurement and were given oral supplementation and resampled before cohort results were analyzed. On that corrected background, 74% (31/42) of patients at first sampling had at least one abnormal measurement of calcium metabolism, defined in this study as pH-corrected ionized calcium, albumin-corrected total calcium, PTH, phosphate, magnesium, 25-hydroxy-vitamin D, alkaline phosphatase and urinary calcium to creatinine ratio. Low or high levels are defined as those outside the hospital diagnostic laboratory age-appropriate reference ranges. The commonest findings were moderately low serum ionized calcium (the active form) in 41% (15/37), high PTH in 17% (7/42), and appropriately adjusted urinary calcium excretion for abnormal serum levels in 17% (5/30) patients. We undertook repeat sampling in 26 and 10 patients (2 and 3 sampling time points, respectively) (Figure 2a; Supplementary Table S1). This demonstrated fluctuating levels of abnormal measurements within patients but with a similar overall proportion of abnormal results in the cohort at each time point (69% and 80% at sampling points 2 and 3, respectively). Mirroring this, expected inter-relationships between parameters, for example inverse levels of serum calcium and of PTH, were not always preserved within an individual at a particular time point, but were clearly related in the normal manner when the cohort measurements were considered as a whole group (Supplementary Figure S1). For example, serum PTH showed the expected inverse correlation with serum calcium (Supplementary Figure S1a), and urinary calcium to creatinine ratio appropriately increased with increasing serum calcium level (Supplementary Figure S1b).

To attempt to unpick these profiles further, we went on to measure intact and C-terminal fibroblast growth factor 23 (iFGF23) and 1,25-dihydroxy-vitamin D in those patients who agreed to repeat testing and in whom adequate sample could be obtained (Supplementary Table S1). C-terminal fibroblast growth factor 23 was high in 9 of 20 patients (mean 105.9 RU/ml, range 23–355) with normal iFGF23 in 17 of 18 and normal 1,25-dihydroxyvitamin D and phosphate concentrations. Notably, C-terminal fibroblast growth factor 23 and iFGF23 levels showed an opposite correlation with different physiological parameters, and only iFGF23 displayed statistically significant negative correlation with 1,25-dihydroxyvitamin D (Supplementary Figure S1). In 7 of 20 patients, 1,25-dihydroxyvitamin D was low (mean 129.6 pmol/l, range 53–218), all with normal 25-hydroxy-vitamin D levels (Supplementary Table S1). iFGF23 and 1,25-dihydroxyvitamin D showed the expected inverse correlation (Supplementary Figure S1c), whereas no correlation was observed between 1,25-dihydroxyvitamin D and PTH (Supplementary Figure S1d).

### Patients with SWS/PPV have no major abnormalities of calcium metabolic functioning of parathyroids, kidneys, and skeletal systems

**Supplementary Figure S2:**
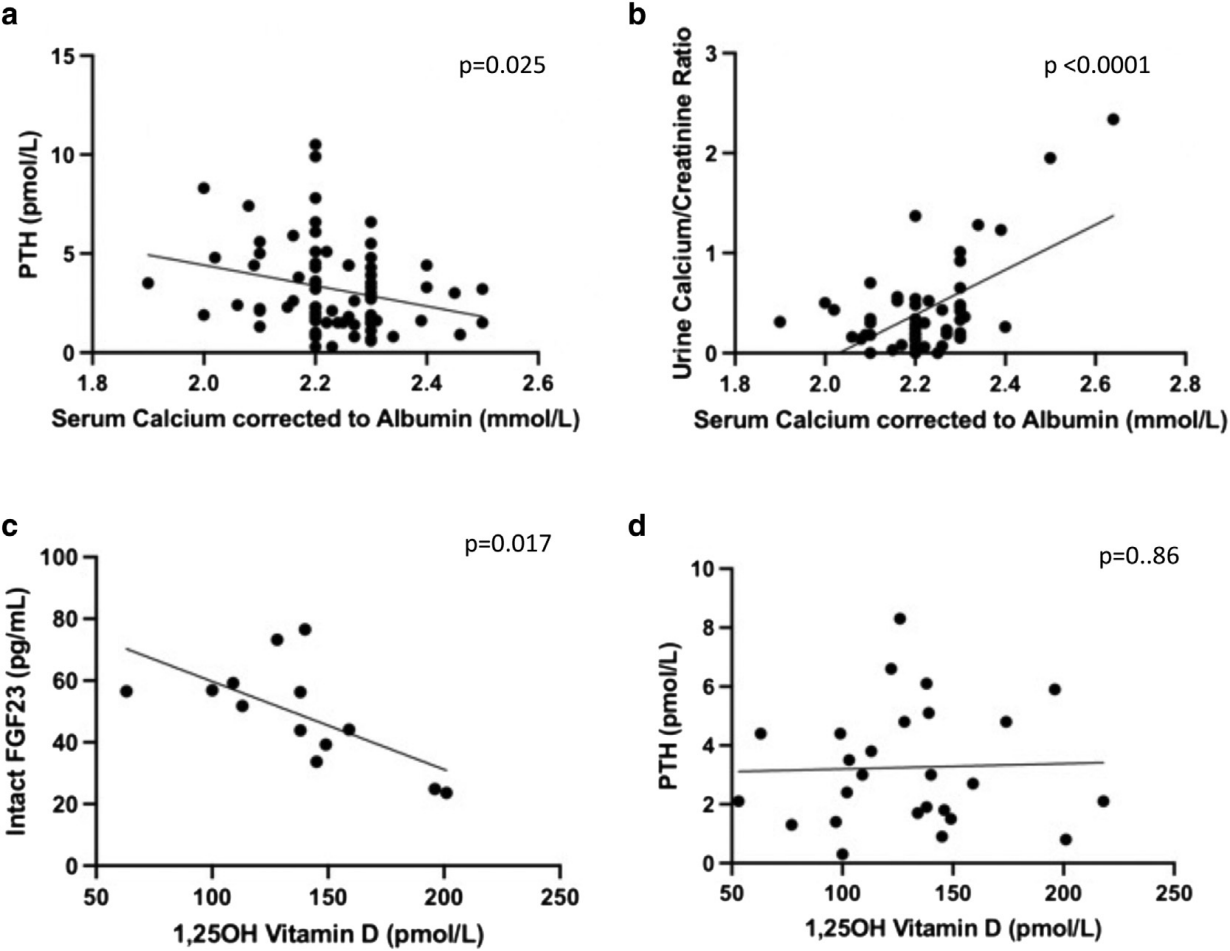
**Calcium homeostatic relationships governed by the parathyroid and kidney behave normally in the *GNAQ/GNA11* mosaic cohort.** (**a**) Significant inverse correlation between serum calcium corrected to albumin and serum PTH. (**b**) Significant correlation between serum calcium corrected to albumin and urine calcium/creatinine ratio. (**c**) Significant correlation between 1,25-hydroxyvitamin D and intact FGF23. (**d**) Correlation between 1,25-hydroxyvitamin D and PTH. Statistical correlations were performed in Prism as detailed in the methods. FGF23, fibroblast growth factor 23; 1,25OH, 1,25-hydroxy; PTH, parathyroid hormone.

Owing to the variability in interpatient and intrapatient measurements, associations between key calcium metabolic parameters were modeled at cohort level. PTH showed the expected inverse correlation with serum calcium level (Supplementary Figure S2a), urinary calcium to creatinine ratio appropriately increased with increasing serum calcium level (Supplementary Figure S2b), and iFGF23 and 1,25-dihydroxyvitamin D showed the expected inverse correlation (Supplementary Figure S2c), whereas no correlation was observed between 1,25-dihydroxyvitamin D and PTH (Supplementary Figure S2d). This lack of relationship between PTH and 1,25-dihydroxyvitamin D indicates that iFGF23 may be the physiological regulator of 1,25-dihydroxyvitamin D in these patients. Estimated glomerular filtration rate measurements were normal throughout (Supplementary Table S1), as were blood pressure measurements where available (n = 39). Whole-body DEXA scans were normal in 11 patients with hypocalcemia and borderline abnormal when excluding the head in 1 patient (Z score = −1.9).

### Serum-ionized calcium is significantly inversely associated with seizures, status epilepticus, and antiepileptics

Multiple linear regression modeling of total serum corrected calcium showed a significant negative association with increasing age (*P* = .001) (Figure 2b) and no association with affected skin surface area. Linear regression of urinary calcium to creatinine ratio by age alone showed the same significant negative association with increasing age (*P* = .001), as did serum magnesium and phosphate (*P* < .001 both).

We then modeled the commonest adverse outcome, patient seizures, using the commonest serum abnormality, ionized calcium. This demonstrated a significant inverse association (*P* = .013) between serum pH-corrected ionized calcium level and the presence of seizures (Figure 2c), and of status epilepticus (*P* = .017) (Figure 2d), independent of the affect of age. Significant associations between ionized calcium and levetiracetam (*P* = .02) and oxcarbazepine (*P* = .003) use were also seen (corrected for age in the multiple regression) but not with other antiepileptics (Supplementary Table S2). No association between prophylactic aspirin use and occurrence of seizures was found in this cohort.

For the key abnormal calcium metabolic parameters patient sex did not significantly affect the regression findings. The statistical contribution of different diagnostic labels and of genotype were not modeled given the cohort size in these rare diseases, but could be of interest in the future.

### Histopathology of affected brain sections demonstrates intravascular, perivascular and intraparenchymal mineral deposition, and primarily microvascular disease

**Figure 3:**
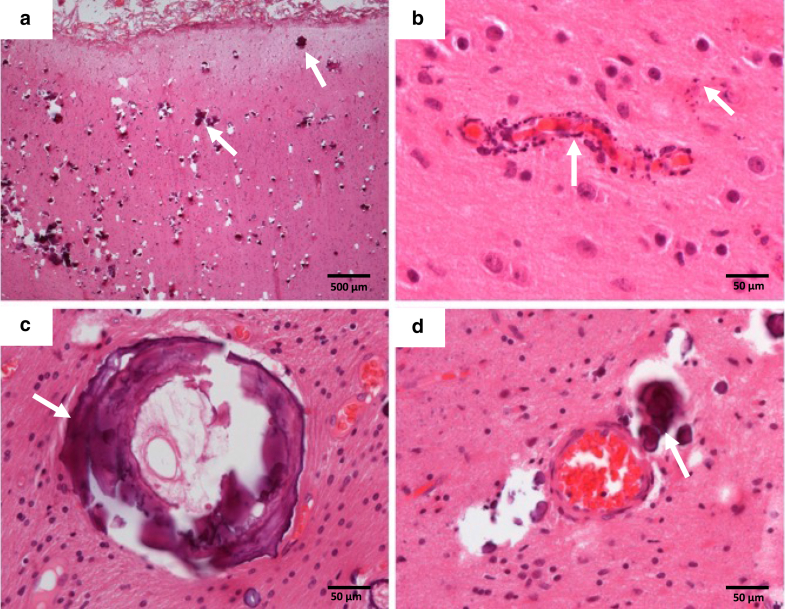
**Localized intravascular, perivascular, and parenchymal patterns of mineral (calcium) deposition.** (**a**) Image of the cortex with extensive foci of mineralization. (**b**) A small cortical vessel (likely to be a capillary) with granular mineralization of the wall. (**c**) A white matter vessel encircled by mineral and fibrosis. (**d**) A white matter vessel with perivascular deposits and granular parenchymal mineral deposits. In each image, the arrow indicates an example of the mineral deposits. The scale bar represents 500 micrometres (**a**) and 50 micrometres (**b–d**).

Histological sections of cerebral cortex and overlying leptomeninges from five cases of epilepsy surgery were reviewed. All patients had calcification on previous brain imaging. All showed abnormal mineral deposits (Figure 3a–d), classified as very extensive in four and sparse in one, affecting the cortical parenchyma in all, the white-matter parenchyma in four, and the leptomeninges in one patient. There were frequent perivascular deposits (Figure 3b and d), but there was also genuine mineralization of the walls of both very small (mostly cortical) vessels (presumed capillaries) and small venules or arterioles (usually in the white matter) (Figure 3b and c). In two cases, the latter was quite extensive with the vessel encircled by dense mineral. The location of the mineral in the wall was not clear, but in those vessels in which a distinction was possible, it appeared to be in the tunica adventitia and media.

In addition, the cases showed typical vascular malformations within the leptomeninges, and two cases showed focal disruption of the normal cortical architecture, a feature previously recognized in some patients with SWS. Additional nonspecific neuropathological findings included gliosis and volume loss, demonstrated by sulcal widening.

## Discussion

This study began with a reappraisal of the potential pathogenetic relevance of neurovascular calcification as one of the cardinal signs of disease in *GNAQ/GNA11* mosaicism. We have demonstrated clear, although moderate, abnormalities of calcium metabolic profile in the cohort as a whole, with a tendency towards ionised hypocalcemia. On the basis of normal parathyroids and renal function, we considered that this could be related to taking antiseizure medications, which are commonly understood to cause 25-hydroxy-vitamin D–deficiency and resultant calcium metabolic abnormalities (Aksoy et al, 2016; Koo et al, 2013; Nissen-Meyer et al, 2007; Verrotti et al, 2010). In support of this, we identified a significant association between two commonly used antiseizure medications, levetiracetam and oxcarbazepine, and lower (although not necessarily abnormal) calcium levels. Such a relationship has previously been described in (non-*GNAQ/GNA11*) patients with seizures (Aksoy et al, 2016).However again those patients had low 25-hydroxy-vitamin D, which our patient cohort did not have. There is, therefore, either an as yet unknown explanation for their calcium metabolic disturbances, potentially related to the calcium signaling disturbances, or the antiseizure drug effect on calcium metabolic profiling is not only mediated through abnormal 25-hydroxy-vitamin D levels. Independent of the mechanism, the systemic findings may provide an important and to our knowledge previously unreported insight for clinical management of these patients with deteriorating neurology in the first year of life. Calcium is known as a stabilizer of excitable membranes, and although the decreased levels of serum calcium would not be expected to cause seizures in a healthy individual, in the context of a seizure disorder they could be a contributory factor. Furthermore, owing to the demonstrable cellular abnormalities reported in Zecchin and Knöpfel et al (2024), local levels of extracellular perivascular calcium may be much more profoundly depleted, which could have direct local effects on cells in the region of the vascular malformations. At biological level the interaction between severely abnormal cells and normal cell populations in a mosaic disorder can produce unpredictable effects and in this regard mosaic disorders lie closer to tumor-host interactions than to germline monogenic diseases.

Histopathological review in this study confirms widespread mineral deposits throughout the cortex and white matter, the vascular and perivascular nature of many of the deposits, and the additional presence of intraparenchymal deposits not clearly related to vessels. Whether this is due to obliteration of previous small vessels through the calcification process or to primary parenchymal calcification is not known. Importantly, where the parenchymal calcification is related to vasculature, this is largely microvascular, and therefore similar in nature to the microvascular disease in the skin. This adds an important insight into Sturge-Weber and PPV-DM neurovascular disease, previously considered to be related only to larger vessel abnormalities and their effects on underlying cerebral perfusion. Occlusion of microvasculature by progressive mineral deposition could be a critical component of the abnormal cerebral perfusion and the postnatal neurodeterioration typical of the disease. If this is the case, early therapeutic intervention to prevent microvascular calcification could potentially be extremely important, even in the context of irreversible larger vessel malformations.

## Materials and Methods

### Patient cohort

Forty patients with a clinical diagnosis of SWS or PPV-DM and two affected by extensive capillary malformation only were recruited prospectively from a single center with written informed consent by their parents or guardians and under local Research Ethics Committee approval (London Bloomsbury Research Ethics Committee of Great Ormond Street Hospital/UCL Institute of Child Health, London, United Kingdom). Patients and/or parents/guardians provided written consent for the publication of images. Patients with SWS and PPV-DM have been grouped together as they are part of the spectrum of *GNAQ/GNA11* mosaic disorders and have an identical vascular phenotype and associated clinical phenotype. Clinical and radiological phenotyping of cutaneous, neurological, and ophthalmological manifestations and calcium metabolic profile analysis in blood and urine were undertaken.

Cutaneous features recorded were the presence or absence of capillary malformation (port wine stain with or without naevus anaemicus), dermal melanocytosis, and involvement of the forehead area by vascular and/or pigmentary lesions. The proportion of the body covered by the capillary malformation was estimated using the Lund-Browder chart. Other recorded features were head circumference, overgrowth, or undergrowth of other body areas, skeletal and endocrinological abnormalities, blood pressure, and neurological and ophthalmological phenotype. Retrospective review of all brain computed tomography (n = 7) and magnetic resonance imaging (n = 36) studies, including gradient-echo imaging (ie T2∗, susceptibility-weighted imaging or the b0 map of the diffusion-weighted sequence, in case the former were not available), was performed by a single radiologist.

All blood sampling was performed in an out-patient setting while the patient was stable. Blood indices measured were ionized calcium, total calcium, phosphate, magnesium, PTH, alkaline phosphatase, FGF23, both C-terminal fibroblast growth factor 23 and iFGF23 (Immutopics), 1,25-dihydroxyvitamin D, 25-hydroxy-vitamin D, urea, and electrolytes. Urinary calcium to creatinine ratio, tubular reabsorption of phosphate, ratio of tubular maximum reabsorption of phosphate to glomerular filtration rate and estimated glomerular filtration rate were also determined. Patients with serum-ionized hypocalcemia were offered a whole-body DEXA scan, performed in 12 cases. Comparison of serum indices was made by reference to the Great Ormond St Hospital reference laboratory standards (Lockitch et al, 1988).

Genotyping of affected tissue by 4-mm skin punch biopsy was offered to the entire cohort. DNA was extracted by standard methods from whole skin and underwent targeted panel sequencing for all coding sequences of *GNAQ* and *GNA11* to a mean depth of 1500× using Illumina technology.

Expert neurohistopathological review was undertaken of brain sections from five patients involved in the study in whom epilepsy surgery had previously been performed, using H&E and elastin Van Gieson staining.

### Statistical analysis

Multiple linear regression analysis was performed to model serum corrected calcium and urinary calcium to creatinine ratio each by age, sex, and affected skin area, using SPSS v.28, and with *P*-value significance adjusted for multiple testing. Multiple binary regression was performed to model seizures and status epilepticus against the same independent variables. Correlations among serum parameters were modeled in Prism using simple linear regression analysis and stringency (ROUT Q = 1%), which resulted in a single outlier removal from 65 measurements. Removal of the outlier did not alter the significance or otherwise of any of correlations.

### Study approval

The study was approved by the London Bloomsbury Research Ethics Committee of Great Ormond Street Hospital/UCL Institute of Child Health (London, United Kingdom). All participants’ parents or guardians provided written informed consent for skin biopsy and/or brain tissue sampling for genetic testing and blood or urine investigations. Separate written informed consent was obtained for publication of clinical photographs.

## Data availability statement

All data are available in the main text or the supplementary materials.

## ORCIDs

Nicole Knöpfel: http://orcid.org/0000-0002-6438-6550

Davide Zecchin: http://orcid.org/0000-0002-4784-0336

Hanna Richardson: http://orcid.org/0009-0005-5589-8027

Satyamaanasa Polubothu: http://orcid.org/0000-0001-7195-5670

Sara Barberan-Martin: http://orcid.org/0000-0003-0142-4078

Thomas Cullup: http://orcid.org/0009-0009-9850-0698

Karolina Gholam: http://orcid.org/0000-0002-8109-6993

Simon Heales: http://orcid.org/0000-0002-9906-0200

Steve Krywawych: http://orcid.org/0009-0000-9161-5550

Pablo López-Balboa: http://orcid.org/0000-0002-9944-719X

Noreen Muwanga-Nanyonjo: http://orcid.org/0009-0005-4772-4748

Olumide Ogunbiyi: http://orcid.org/0000-0001-5208-5526

Clinda Puvirajasinghe: http://orcid.org/0000-0003-1646-6588

Lea Solman: http://orcid.org/0000-0002-6183-6608

Samira B. Syed: http://orcid.org/0000-0002-3872-5870

Zubair Tahir: http://orcid.org/0000-0001-9797-3770

Martin M. Tisdall: http://orcid.org/0000-0001-8880-8386

Jeremy Allgrove: http://orcid.org/0000-0003-0858-7064

Alexander D. Chesover: http://orcid.org/0000-0002-6280-5053

Sarah E. Aylett: http://orcid.org/0000-0001-9630-3222

Thomas S. Jacques: http://orcid.org/0000-0002-7833-2158

Fadil M. Hannan: http://orcid.org/0000-0002-2975-5170

Ulrike Löbel: http://orcid.org/0000-0001-9844-3464

Robert K. Semple: http://orcid.org/0000-0001-6539-3069

Rajesh V. Thakker: http://orcid.org/0000-0002-1438-3220

Veronica A. Kinsler: http://orcid.org/0000-0001-6256-327

## Conflict of Interest Statement

The authors state no conflict of interest.

## Figures and Tables

**Supplementary Table S1:** Systemic Calcium Metabolic Profiling in Patients with SWS and PPV-DM

Patient No.	Sex	Genotype	Age, years	Ionized calcium	Total calcium	Phosphate	PTH	ALP	25-hydroxy-vitamin D	1,25-dihydroxy-vitamin D	FGF23 (c-terminal fragment)	FGF23 (intact molecule)	Urine Ca/Cr ratio	TRP (%)	TmP/GFR	E-GFR
1	F	*GNAQ* c.548G>A, p.R183Q	3.8	1.23	2.39	1.33	1.6	221	89	-	-	-	Increased	-	-	90.2
			4.8	1.03 (low)	2.00 (low)	1.66	8.3 (high)	177	94	-	69	55.4	-	-	-	107.8
			5.3	1.25	2.30	1.64	1.5	154	95	126	-	-	Normal	85.8	1.41	80.8
2	M	*GNAQ* and *GNA11* WT	3.1	1.24	2.24	1.59	1.5	236	72	-	-	-	-			-
			4.3	1.10 (low)	2.10 (low)	1.88 (high)	5.6	196	107	149	74	39.3	Decreased	91.4	1.72	-
			4.8	1.21	2.20 (low)	1.58	0.3 (low)	164	101	-	-	-	Normal	94.1	1.49	112.8
3	M	*GNAQ* c.548G>A, p.R183Q	16.1	1.22	2.23	1.04	0.3 (low)	146 (high)	53	-	-	-	-	-	-	70.7
			17.2	1.19 (low)	2.20	0.98	1.8	103	76	100 (low)	79	56.9	Normal	95.8	1.28	82.2
4	F	-	4.0	1.22	2.40	1.32	4.4	239	95	-	-	-	Normal	-	-	-
			5.0	1.26	2.30	1.47	5.5	186	105	-	71	-	-	-	-	120.9
5	M	-	15.9	1.24	2.1	0.98	1.3	78	174	-	-	-	Normal	-	-	128.6
			17.4	1.21	2.20	1.04	2.3	76	67	77 (low)	-	-	Normal	91.3	0.95	106.2
6	M	-	2.7	1.22	2.20 (low)	1.54	3.5	109 (low)	79	-	-	-	-	-	-	94.1
7	F	*GNAQ* c.548G>A, p.R183Q	11.0	-	2.30	1.53	4.8	300	57	-	-	-	Normal	-	-	136.1
			12.1	1.19 (low)	2.20	1.54	6.1 (high)	245	107	174	-	-	Normal	93.8	1.44	120.1
			12.4	1.20 (low)	2.20	1.64	3.2	238	85	138	84	43.9	Normal	96.5	1.58	97.36
8	M	*GNAQ* c.548G>A, p.R183Q	1.9	1.24	2.30	1.58	3.0	175	97	-	-		-	-	-	-
			3.1	1.19	2.30	1.78	1.5	417 (high)	83	109	355 (high)	59.2	-	-	-	133.9
9	F	*GNAQ* c.548G>A, p.R183Q	11.8	1.12 (low)	2.16 (low)	1.45	2.6	174	78	-	-	-	Normal	-	-	122.6
10	M	*GNAQ* c.548G>A, p.R183Q	11.6	1.22	2.27	1.34	0.8	306	81	-	-	-	Normal	-	-	114.4
11	M	-	15.0	1.13 (low)	2.09 (low)	1.20	4.4	86 (low)	57	-	-	-	Normal	-	-	127.1
			16.0	1.19 (low)	2.10	1.4	2.1	70	79	63 (low)	63	56.5	Normal	97.2	1.36	137.3
			16.5	1.20	2.20	1.21	2.0	62	-	53 (low)	-	-	Normal	98.3	1.19	100.2
12	M	-	2.4	-	2.27	1.59	1.4	139 (low)	90	-	-	-	-	-	-	122.4
			3.6	1.19	2.20 (low)	1.76	9.9 (high)	140 (low)	117	97 (low)	43	-	Increased	85.8	1.51	73.47
13	F	*GNAQ* c.548G>A, p.R183Q	9.2	1.26	2.45	1.05 (low)	3.0	132	118	-	-	-	-	-	-	125.5
			10.2	1.31	2.5	1.19	3.2	163	125	140	124 (high)	76.5	-	-	-	105.4
			10.5	1.32 (high)	2.4	1.28	3.3	160	105	-	68	82.3	-	-	-	112.3
14	F	*GNAQ* c.548G>A, p.R183Q	9.4	1.17 (low)	2.26	1.57	4.4	128 (low)	66	-	-	-	Normal	-	-	108.9
			10.6	1.25	2.20	1.45	1.0	130	87	99 (low)	-	-	-	-	-	-
15	M	*GNAQ* c.548G>A, p.R183Q	11.8	1.22	2.26	1.29	4.4	171 (low)	111	-	-	-	-	-	-	-
16	M	-	1.4	-	2.46 (high)	1.58	0.9	317	114	-	-	-	-	-	-	-
			2.7	1.22	2.30	1.43	0.6 (low)	259	131	145	23	33.7	-	-	--	83.3
17	F	-	4.0	1.25	2.31	1.52	1.6	217	81	-	-	-	Normal	-	-	92.61
18	F	*GNAQ* c.548G>A, p.R183Q	0.7	1.26	2.64 (high)	1.77	8.9 (high)	171	130	-	-	-	Increased	-	-	-
			1.1	-	2.30	2.21 (high)	-	225	122	-	-	-	-	-	-	97.16
19	M	*GNAQ* c.548G>A, p.R183Q	10.9	1.15 (low)	2.06 (low)	1.37	2.4	198	97	-	-	-	Normal	-	-	121.64
			12.3	1.19 (low)	2.10 (low)	1.74	2.2	103 (low)	51	102 (low)	-	-	Normal	98.9	1.72	148.1
20	M	-	10.8	1.18 (low)	2.25	1.71	1.5	154	62	-	-	-	Decreased	-	-	181.6
			12.1	1.20 (low)	2.20	1.64	0.9	137 (low)	60	-	-	-	Decreased	-	-	133.3
21	M	*GNAQ* and *GNA11* WT	5.5	1.19 (low)	2.20 (low)	1.54	4.5	277	70	-	-	-	Normal	-	-	135.6
22	M	*GNAQ* c.548G>A, p.R183Q	11.2	1.21 (low)	2.22	1.65	1.5	197 (low)	82	-	-	-	Normal	-	-	152.6
23	M	*GNAQ* c.548G>A, p.R183Q	13.7	1.15 (low)	2.08 (low)	1.50	7.4 (high)	353	55	-	-	-	Normal	-	-	105.9
24	F	*GNAQ* c.548G>A, p.R183Q	10.3	1.25	2.15 (low)	1.30	2.3	279	97	-	-	-	Decreased	-	-	122.12
25	F	-	9.6	1.14 (low)	2.02 (low)	1.46	4.8	205	64	-	-	-	Normal	-	-	137.5
			10.5	0.78 (low)	1.90 (low)	2.00 (high)	3.5	230	88	128	35	73.2	Normal	93.8	1.88	114.9
			10.9	1.15 (low)	2.0 (low)	1.51	1.9	302	80	103 (low)	-	-	Normal	92.1	1.39	111.9
26	F	*GNAQ* c.548G>A, p.R183Q	6.00	1.24	2.23	1.56	2.1	359	60		-	-	Normal	-	-	146.0
			6.8	1.28	2.30	1.49	3.3	462 (high)	73	218	-	-	Normal	96.5	1.44	159.56
27	F	*GNAQ* c.548G>A, p.R183Q	2.4	1.14 (low)	2.16 (low)	1.62	5.9 (high)	226	132		-	-	Normal	-	-	-
			3.3	1.19	2.20 (low)	1.49	5.1	253	135	196	167 (high)	24.9	-	-	-	120.21
			3.8	1.25	2.20 (low)	1.49	0.8	258	116	139	-	-	Normal	97.6	1.38	-
28	F	-	15.4	1.11 (low)	2.20	1.38	2.30	66	66	-	163 (high)	-	-	-	-	100.2
29	F	-	1.3	1.29	2.30	1.56	3.9	125	85	-	137 (high)	50.9	-	-	-	102.1
30	M	*GNA11* c.547C>T, p.R183C	5.9	1.19 (low)	2.20 (low)	1.81 (high)	3.6	221	80	-	-	-	-	-	-	113.2
			6.7	1.24	2.20 (low)	1.32	1.6	141 (low)	121	-	-	-	Normal	93.8	1.24	148.6
31	M	*GNAQ* c.548G>A, p.R183Q	4.3	1.12 (low)	-	-	6.6 (high)	-	99	-	-	-	Normal	-	-	-
32^1^	F	*GNA11* c.547C>T, p.R183C	10.5	1.27	2.34	1.29	0.8	248	103	-	-	-	Increased	-	-	112.6
			11.5	-	2.30	1.34	4.3	157	125	201	110 (high)	23.6 (low)	Normal	94.1	1.26	97.3
33^1^	F	*GNA11* c.547C>T, p.R183C	8.4	1.24	2.30	1.45	1.8	260	52	-	-		Normal	-	-	91.7
			9.1	1.22	2.30	1.38	1.1	228	68	146	-	-	Normal	86.9	1.2	86.1
34^1^	F	*GNAQ* c.548G>A, p.R183Q	7.6	1.22	2.26	1.56	1.8	170 (low)	65	-	-	-	Normal	-	-	72.5
35^1^	F	*GNAQ* and *GNA11* WT	14.3	-	2.20	1.23	7.8 (high)	83	58	-	-	-	Normal	-	-	88.8
			14.7	1.20 (low)	2.20	1.31	10.5 (high)	68	35 (low)	-	-	-	-	-	-	93.2
			15.7	1.24	2.10	1.47 (high)	5.0	61	71	-	-	-	Normal	91.4	1.34	90.6
36	F	*GNA11* c.547C>T, p.R183C	6.3	1.18 (low)	2.27	1.58	2.6	218	64	-	-	-	Normal	-	-	103.4
			7.2	1.23	2.30	1.47	2.7	163 (low)	52	-	-	-	-	-	-	116.8
			7.7	1.30	2.30	1.38	3.5	197	71	159	53	44.1	Increased	90.2	1.24	117.6
37^1^	M	*GNA11* c.547C>T, p.R183C	3.8	-	2.22	1.92 (high)	5.1	193	50	-	-	-	Normal	-	-	-
			4.7	1.26	2.30	1.92 (high)	1.7	166	98	-	137 (high)	-	Normal		-	129.7
			5.1	1.19 (low)	2.30	1.83 (high)	0.7	161	76	134	66	-	Normal	87.5	1.6	109.5
38^1^	M	*GNAQ* c.548G>A, p.R183Q	12.6	1.18 (low)	2.17 (low)	1.65	3.8	219	75	-	-	-	Normal	-	-	120.8
			13.6	1.22	2.2	1.46	4.3	203	83	113	74	51.8	Normal	91.6	1.34	106.1
39	F	*GNAQ* c.548G>A, p.R183Q	2.0	1.28	2.30	1.66	2.8	185	141	-	134 (high)	52.5	-	-	-	102.0
40	M	*GNA11* c.547C>T, p.R183C	9.8	1.29	2.30	1.53	6.6 (high)	215	92	-	200 (high)	39.0	Normal	-	-	110.7
			10.4	1.26	2.30	1.41	1.9	230	84	122	-	-	Normal	94.2	1.33	108.7
41	F	-	1.5	1.30	2.20	1.96	6.6 (high)	129 (low)	116	138	-	56.3	Normal	86.2	1.69	109.5
42	M	-	0.2	1.39	2.50 (high)	2.21 (high)	1.50	130	99	-	-	-	Normal	86.9	1.92	-

Abbreviations: cFGF23, C-terminal fragment fibroblast growth factor 23; E-GFR, estimated glomerular filtration rate; iFGF23, intact molecule fibroblast growth factor 23; PTH, Parathyroid hormone; TmP/GFR, ratio of tubular maximum reabsorption of phosphate to glomerular filtration rate; TRP, intact tubular reabsorption of phosphate; WT, wild-type.

Repeat measurements from the same patient are shown on a darker background. All statistical testing was done on values post Vitamin D supplementation and normalization in the few cases where this was required. Ionized calcium was corrected to pH and total calcium to Albumin. Age-adjusted ionized calcium reference ranges: 1.15–1.41 mmol/L (< 2y), 1.19–1.37 mmol/L (2–5y), 1.22–1.31 mmol/L (5–15y). Age-adjusted total calcium reference ranges: 1.96–2.66 mmol/L (0–5d), 2.17–2.44 mmol/L (5d–3y), 2.22–2.51 mmol/L (3–10y), 2.19–2.66 mmol/L (10–15y), 2.10–2.55 mmol/L (>15y). Age-adjusted phosphate reference ranges: 1.5–2.6 mmol/L (0–5d), 1.2-2.1 mmol/L (5d–3y), 1.2–1.8 mmol/L (3–10y), 1.1–1.75 mmol/L (10–15y), 0.8–1.45 mmol/L (>15y). PTH reference range: 0.7–5.6 pmol/L. Age- and sex-adjusted ALP reference ranges: Female, 65–270 U/L (1–7d), 65–365 U/L (7d–1m), 80–425 U/L (1–3m), 80–345 U/L (3–6m); 60–330 U/L (6–12m), 145–320 U/L (1–3y), 150–380 U/L (3–6y), 175–420 U/L (6–9y), 130–560 U/L (9–11y), 105–420 U/L (11–13y), 70–230 U/L (13–15y), 30–126 U/L (>15y). Male 65–270 U/L (1–7d), 65–365 U/L (7d–1m), 80–425 U/L (1–3m), 80–345 U/L (3–6m); 60–330 U/L (6–12m), 145–320 U/L (1–3y), 150–380 U/L (3–6y), 175–420 U/L (6–9y), 135–530 U/L (9–11y), 200–495 U/L (11–13y), 130–525 U/L (13–15y), 30–126 U/L (>15y). 25-hydroxy-vitamin D reference range: insufficiency (25–50 nmol/L), deficiency (<25 nmol/L). 1,25-dihydroxy-vitamin D reference range: 108–246 pmol/L. FGF23 (C-terminal fragment) reference range: < 100 RU/ml. FGF23 (intact molecule) reference range: 33–100 pg/ml. TRP reference range: 70–100%. Age-adjusted TmP/GFR reference ranges: 1.55–2.97 mmol/L (newborns), 1.07–2.23 mmol/L (1m–2y), 1.10–1.88 mmol/L (2–12y), 0.93–1.71 mmol/L (12–16y), and 0.88–1.26 mmol/L (>16y). EGFR for children and adolescents using Bedside Schwartz Formula (ml/min per 1.73m^2^).

1 indicates a patient previously reported in Polubothu et al. (2020).

**Supplementary Table S2:** Systemic Calcium Metabolic Profiling in Patients with SWS and PPV-DM and Antiepileptic Treatment

Patient No.	Sex	Genotype	Age, years	Ionized calcium	Total calcium	Phosphate	PTH	25-hydroxy-vitamin D	1,25-dihydroxy-vitamin D	FGF23 (c-terminal fragment)	FGF23 (intact molecule)	Urine Ca/Cr ratio	Antiepileptics
1	F	*GNAQ* c.548G>A, p.R183Q	3.8	1.23	2.39	1.33	1.6	89	-	-	-	Increased	Levetiracetam, zonisamide
			4.8	1.03 (low)	2.00 (low)	1.66	8.3 (high)	94	-	69	55.4	-	
			5.3	1.25	2.30	1.64	1.5	95	126	-	-	Normal	
2	M	*GNAQ* and *GNA11* WT	3.1	1.24	2.24	1.59	1.5	72	-	-	-	-	Levetiracetam, sodium valproate, zonisamide
			4.3	1.10 (low)	2.10 (low)	1.88 (high)	5.6	107	149	74	39.3	Decreased	
			4.8	1.21	2.20 (low)	1.58	0.3 (low)	101	-	-	-	Normal	
3	M	*GNAQ* c.548G>A, p.R183Q	16.1	1.22	2.23	1.04	0.3 (low)	53	-	-	-	-	None
			17.2	1.19 (low)	2.20	0.98	1.8	76	100 (low)	79	56.9	Normal	
4	F	-	4.0	1.22	2.40	1.32	4.4	95	-	-	-	Normal	None
			5.0	1.26	2.30	1.47	5.5	105	-	71	-	-	
5	M	-	15.9	1.24	2.1	0.98	1.3	174	-	-	-	Normal	Carbamazepine
			17.4	1.21	2.20	1.04	2.3	67	77 (low)	-	-	Normal	
6	M	-	2.7	1.22	2.20 (low)	1.54	3.5	79	-	-	-	-	Levetiracetam
7	F	*GNAQ* c.548G>A, p.R183Q	11.0	-	2.30	1.53	4.8	57	-	-	-	Normal	Levetiracetam, oxcarbazepine
			12.1	1.19 (low)	2.20	1.54	6.1 (high)	107	174	-	-	Normal	
			12.4	1.20 (low)	2.20	1.64	3.2	85	138	84	43.9	Normal	
8	M	*GNAQ* c.548G>A, p.R183Q	1.9	1.24	2.30	1.58	3.0	97	-	-	-	-	Levetiracetam
			3.1	1.19	2.30	1.78	1.5	83	109	355 (high)	59.2	-	
9	F	*GNAQ* c.548G>A, p.R183Q	11.8	1.12 (low)	2.16 (low)	1.45	2.6	78	-	-	-	Normal	Levetiracetam, topiramate
10	M	*GNAQ* c.548G>A, p.R183Q	11.6	1.22	2.27	1.34	0.8	81	-	-	-	Normal	Levetiracetam, sodium valproate
11	M	-	15.0	1.13 (low)	2.09 (low)	1.20	4.4	57	-	-	-	Normal	Levetiracetam, sodium valproate, gabapentin
			16.0	1.19 (low)	2.10	1.4	2.1	79	63 (low)	63	56.5	Normal	
			16.5	1.20	2.20	1.21	2.0	-	53 (low)	-	-	Normal	
12	M	-	2.4	-	2.27	1.59	1.4	90	-				Topiramate, clobazam
			3.6	1.19	2.20 (low)	1.76	9.9 (high)	117	97 (low)	43	-	Increased	
13	F	*GNAQ* c.548G>A, p.R183Q	9.2	1.26	2.45	1.05 (low)	3.0	118	-	-	-	-	Levetiracetam, sodium valproate
			10.2	1.31	2.5	1.19	3.2	125	140	124 (high)	76.5	-	
		25.10.22	10.5	1.32 (high)	2.4	1.28	3.3	105	-	68	82.3	-	
14	F	*GNAQ* c.548G>A, p.R183Q	9.4	1.17 (low)	2.26	1.57	4.4	66	-	-	-	Normal	None
			10.6	1.25	2.20	1.45	1.0	87	99 (low)	-	-	-	
15	M	*GNAQ* c.548G>A, p.R183Q	11.8	1.22	2.26	1.29	4.4	111	-	-	-	-	Valproate, carbamazepine
16	M	-	1.4	-	2.46 (high)	1.58	0.9	114	-	-	-	-	Levetiracetam, oxcarbazepine, topiramate
			2.7	1.22	2.30	1.43	0.6 (low)	131	145	23	33.7	-	
17	F	-	4.0	1.25	2.31	1.52	1.6	81	-	-	-	Normal	None
18	F	*GNAQ* c.548G>A, p.R183Q	0.7	1.26	2.64 (high)	1.77	8.9 (high)	130	-	-	-	Increased	Levetiracetam, sodium valproate, oxcarbazepine, topiramate
			1.1	-	2.30	2.21 (high)	-	122	-	-	-	-	
19	M	*GNAQ* c.548G>A, p.R183Q	10.9	1.15 (low)	2.06 (low)	1.37	2.4	97	-	-	-	Normal	Levetiracetam, carbamazepine
			12.3	1.19 (low)	2.10 (low)	1.74	2.2	51	102 (low)	-	-	Normal	Levetiracetam
20	M	-	10.8	1.18 (low)	2.25	1.71	1.5	62	-	-	-	Decreased	Levetiracetam
		24.11	12.1	1.20 (low)	2.20	1.64	0.9	60	-	-	-	Decreased	
21	M	*GNAQ* and *GNA11* WT	5.5	1.19 (low)	2.20 (low)	1.54	4.5	70	-	-	-	Normal	None
22	M	*GNAQ* c.548G>A, p.R183Q	11.2	1.21 (low)	2.22	1.65	1.5	82	-	-	-	Normal	None
23	M	*GNAQ* c.548G>A, p.R183Q	13.7	1.15 (low)	2.08 (low)	1.50	7.4 (high)	55	-	-	-	Normal	Oxcarbazepine, briveracetam
24	F	*GNAQ* c.548G>A, p.R183Q	10.3	1.25	2.15 (low)	1.30	2.3	97	-	-	-	Decreased	Carbamazepine
25	F	-	9.6	1.14 (low)	2.02 (low)	1.46	4.8	64	-	-	-	Normal	Oxcarbazepine, zonisamide
			10.5	0.78 (low)	1.90 (low)	2.00 (high)	3.5	88	128	35	73.2	Normal	
			10.9	1.15 (low)	2.0 (low)	1.51	1.9	80	103 (low)	-	-	Normal	
26	F	*GNAQ* c.548G>A, p.R183Q	6.00	1.24	2.23	1.56	2.1	60	-	-	-	Normal	Levetiracetam
			6.8	1.28	2.30	1.49	3.3	73	218	-	-	Normal	
27	F	*GNAQ* c.548G>A, p.R183Q	2.4	1.14 (low)	2.16 (low)	1.62	5.9 (high)	132	-	-	-	Normal	Levetiracetam, oxcarbazepine, topiramate
			3.3	1.19	2.20 (low)	1.49	5.1	135	196	167 (high)	24.9	-	
			3.8	1.25	2.20 (low)	1.49	0.8	116	139	-	-	Normal	Levetiracetam, oxcarbazepine
28	F	-	15.4	1.11 (low)	2.20	1.38	2.30	66	-	163 (high)	-	-	Levetiracetam
29	F	-	1.3	1.29	2.30	1.56	3.9	85	-	137 (high)	50.9	-	None
30	M	*GNA11* c.547C>T, p.R183C	5.9	1.19 (low)	2.20 (low)	1.81 (high)	3.6	80	-	-	-	-	None
			6.7	1.24	2.20 (low)	1.32	1.6	121	-	-	-	Normal	
31	M	*GNAQ* c.548G>A, p.R183Q	4.3	1.12 (low)	-	-	6.6 (high)	99	-	-	-	Normal	Levetiracetam, oxcarbazepine, zonisamide
32^1^	F	*GNA11* c.547C>T, p.R183C	10.5	1.27	2.34	1.29	0.8	103	-	-	-	Increased	None
			11.5	-	2.30	1.34	4.3	125	201	110 (high)	23.6 (low)	Normal	
33^1^	F	*GNA11* c.547C>T, p.R183C	8.4	1.24	2.30	1.45	1.8	52	-	-	-	Normal	Levetiracetam, topiramate
			9.1	1.22	2.30	1.38	1.1	68	146	-	-	Normal	
34^1^	F	*GNAQ* c.548G>A, p.R183Q	7.6	1.22	2.26	1.56	1.8	65	-	-	-	Normal	None
35^1^	F	*GNAQ* and *GNA11* WT	14.3	-	2.20	1.23	7.8 (high)	58	-	-	-	Normal	None
			14.7	1.20 (low)	2.20	1.31	10.5 (high)	35 (low)	-	-	-	-	
			15.7	1.24	2.10	1.47 (high)	5.0	71	-	-	-	Normal	
36	F	*GNA11* c.547C>T, p.R183C	6.3	1.18 (low)	2.27	1.58	2.6	64	-	-	-	Normal	None
			7.2	1.23	2.30	1.47	2.7	52	-	-	-	-	
			7.7	1.30	2.30	1.38	3.5	71	159	53	44.1	Increased	
37^1^	M	*GNA11* c.547C>T, p.R183C	3.8	-	2.22	1.92 (high)	5.1	50	-	-	-	Normal	None
			4.7	1.26	2.30	1.92 (high)	1.7	98	-	137 (high)	-	Normal	
			5.1	1.19 (low)	2.30	1.83 (high)	0.7	76	134	66	-	Normal	
38^1^	M	*GNAQ* c.548G>A, p.R183Q	12.6	1.18 (low)	2.17 (low)	1.65	3.8	75	-	-	-	Normal	None
			13.6	1.22	2.2	1.46	4.3	83	113	74	51.8	Normal	
39	F	*GNAQ* c.548G>A, p.R183Q	2.0	1.28	2.30	1.66	2.8	141	-	134 (high)	52.5	-	None
40	M	*GNA11* c.547C>T, p.R183C	9.8	1.29	2.30	1.53	6.6 (high)	92	-	200 (high)	39.0	Normal	None
			10.4	1.26	2.30	1.41	1.9	84	122	-	-	Normal	
41	F	-	1.5	1.30	2.20	1.96	6.6 (high)	116	138	-	56.3	Normal	None
42	M	-	0.2	1.39	2.50 (high)	**2.21 (high)**	1.50	99	-	-	-	Normal	None

Abbreviations: cFGF23, C-terminal fragment fibroblast growth factor 23; E-GFR, estimated glomerular filtration rate; iFGF23, intact molecule fibroblast growth factor 23; PTH, Parathyroid hormone; TmP/GFR, ratio of tubular maximum reabsorption of phosphate to glomerular filtration rate; TRP, intact tubular reabsorption of phosphate; WT, wild-type.

1 indicates a patient previously reported in Polubothu et al. (2020).

## Supplementary Materials

*GNAQ/GNA11* mosaicism is associated with abnormal serum calcium indices and microvascular neurocalcification.
